# Supplementary figures and images for: High Seroprevalence of SARS-CoV-2 in Mwanza, Northwestern Tanzania: A Population-Based Survey
Source: Int J Environ Res Public Health. 2022 Sep 16;19(18):11664. doi: 10.3390/ijerph191811664 (PMC9517516; doi:10.3390/ijerph191811664)

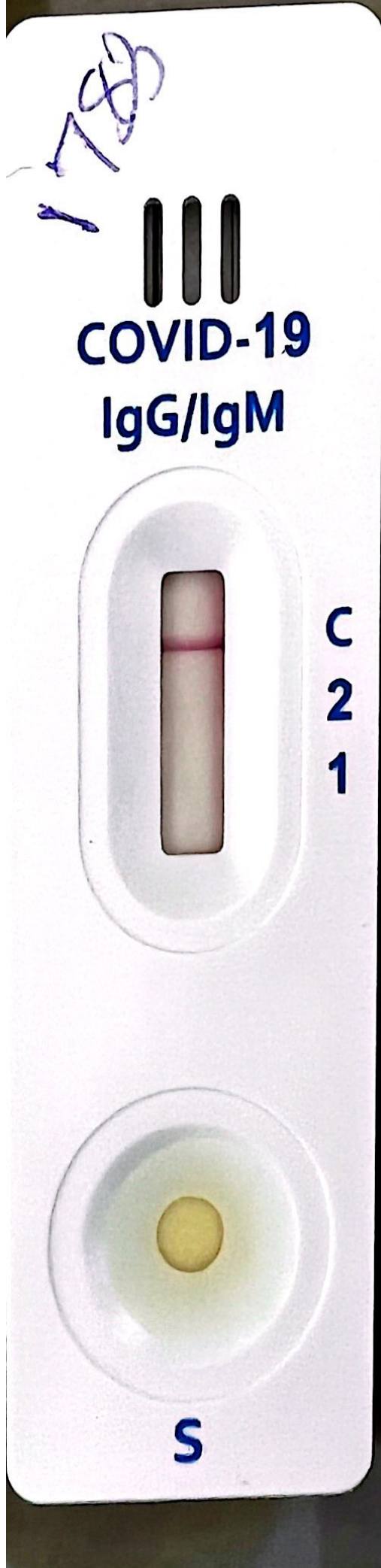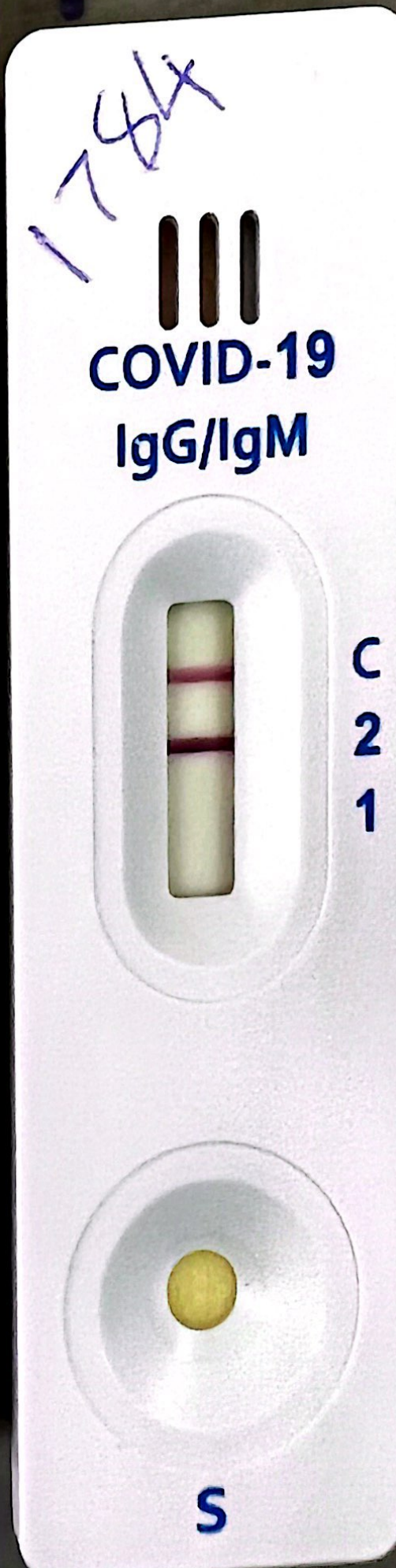

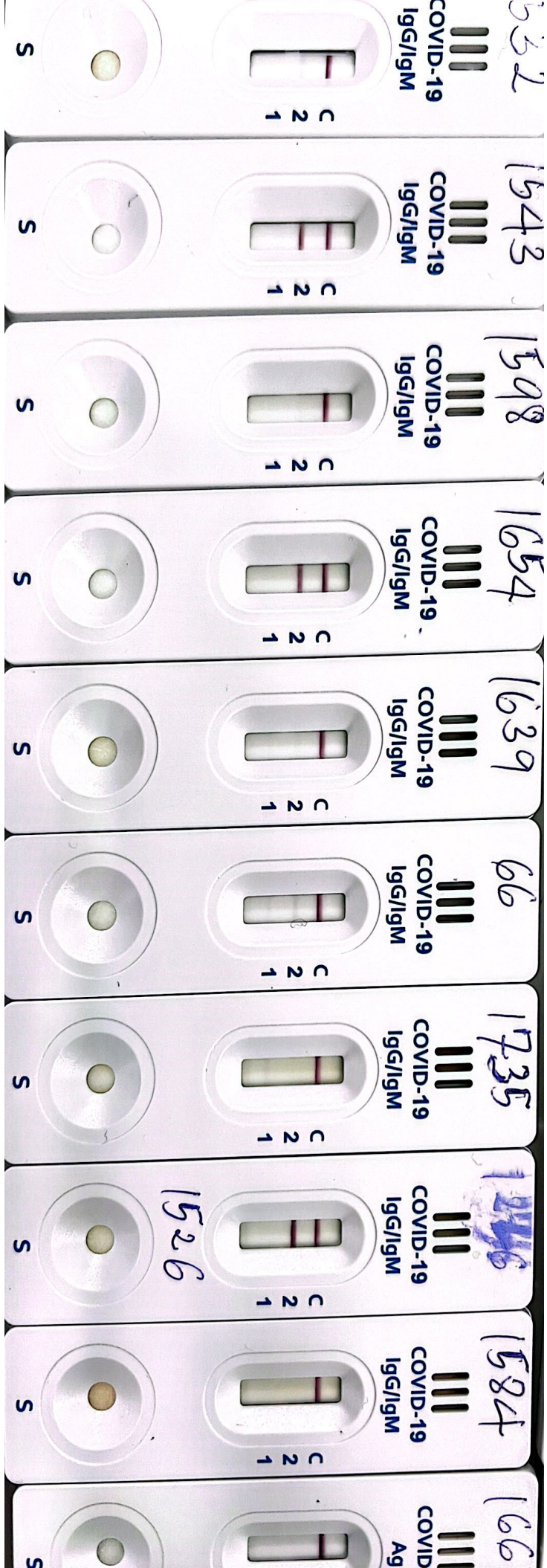

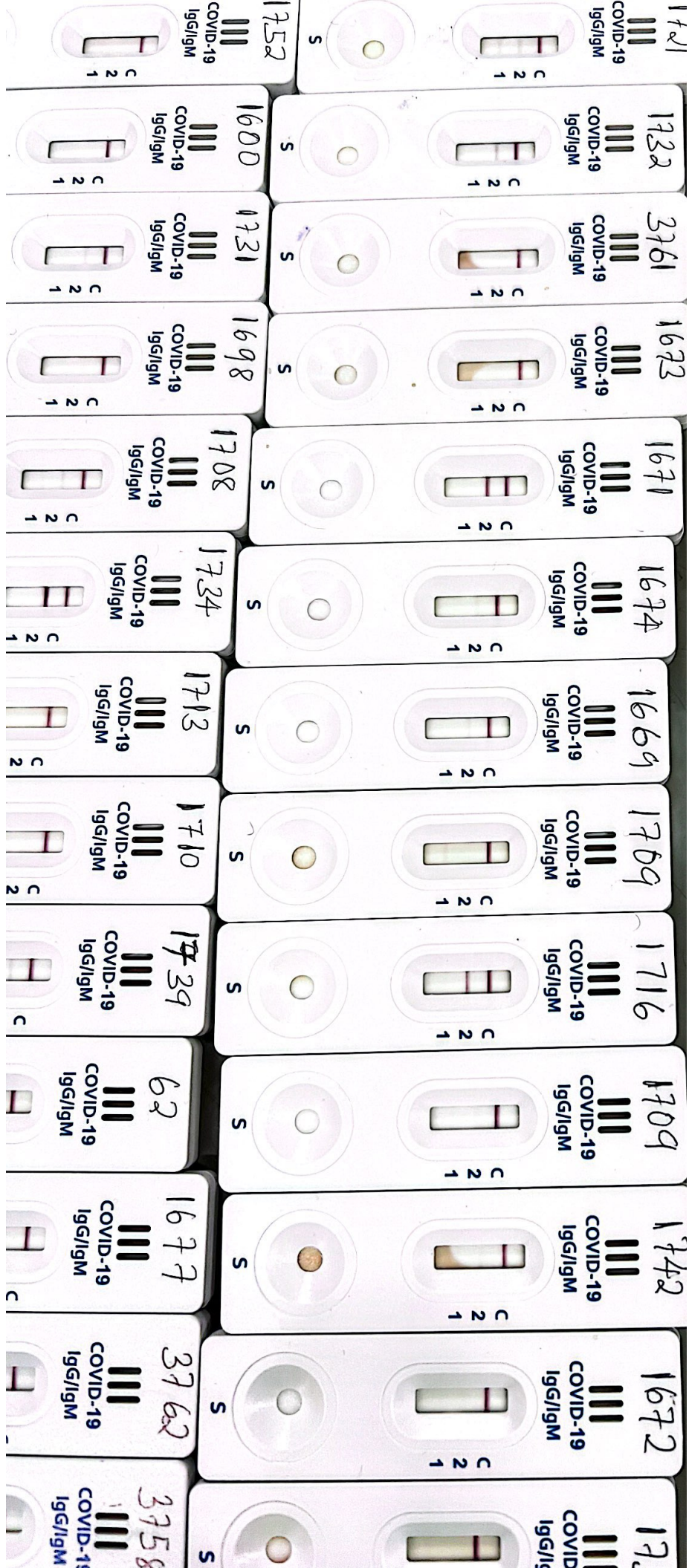

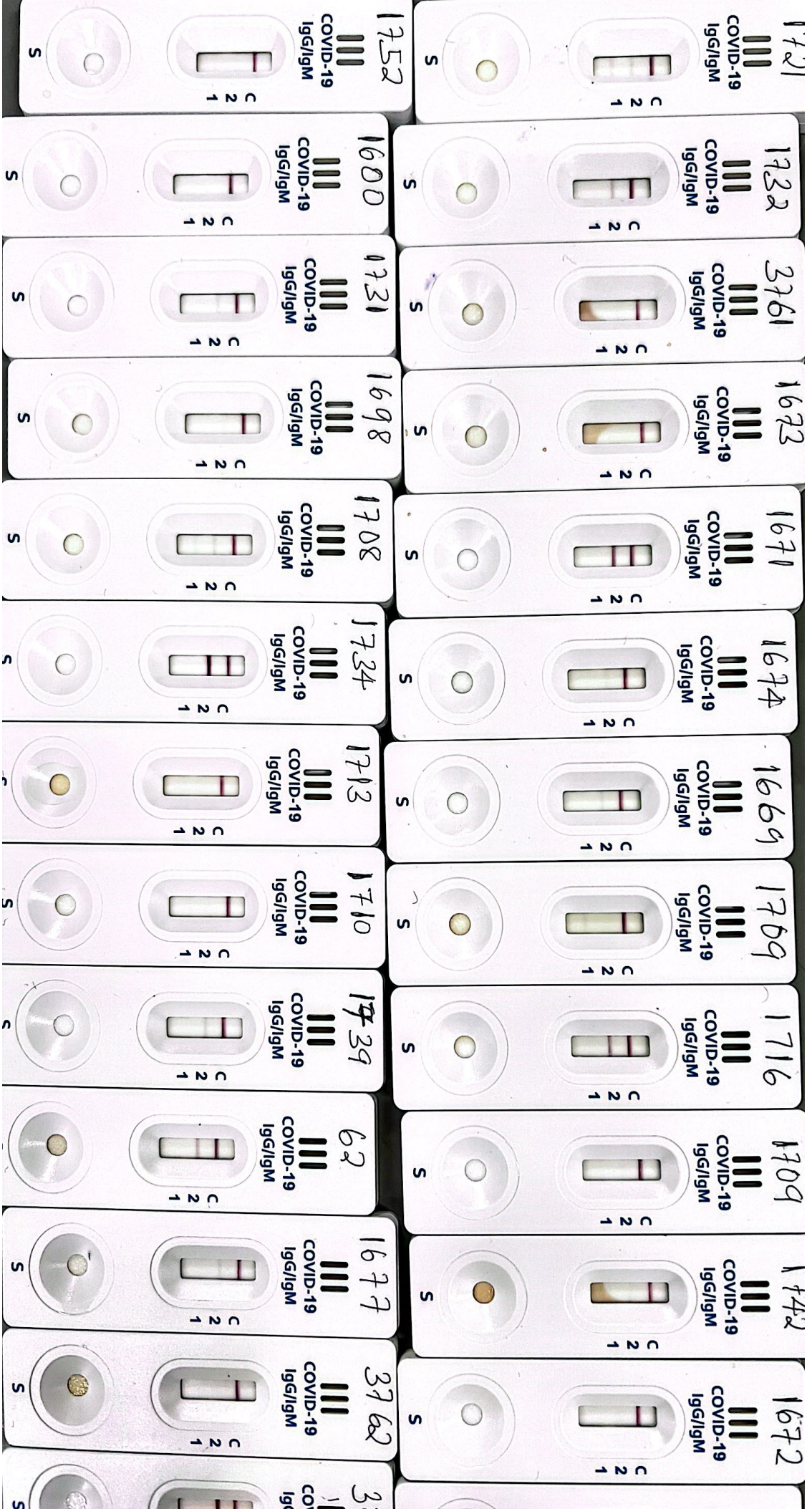

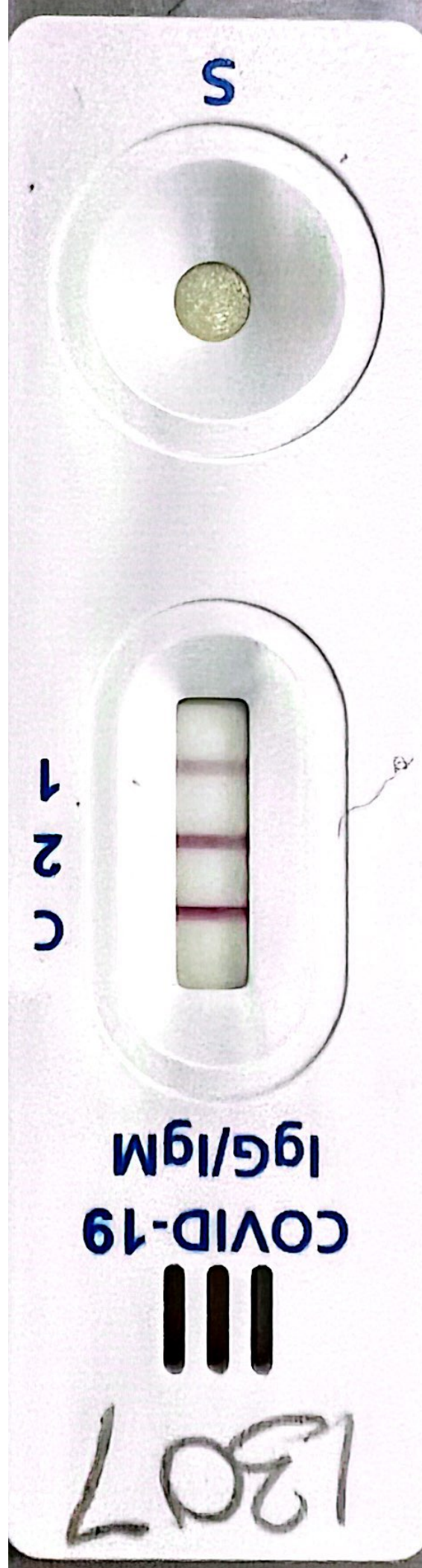

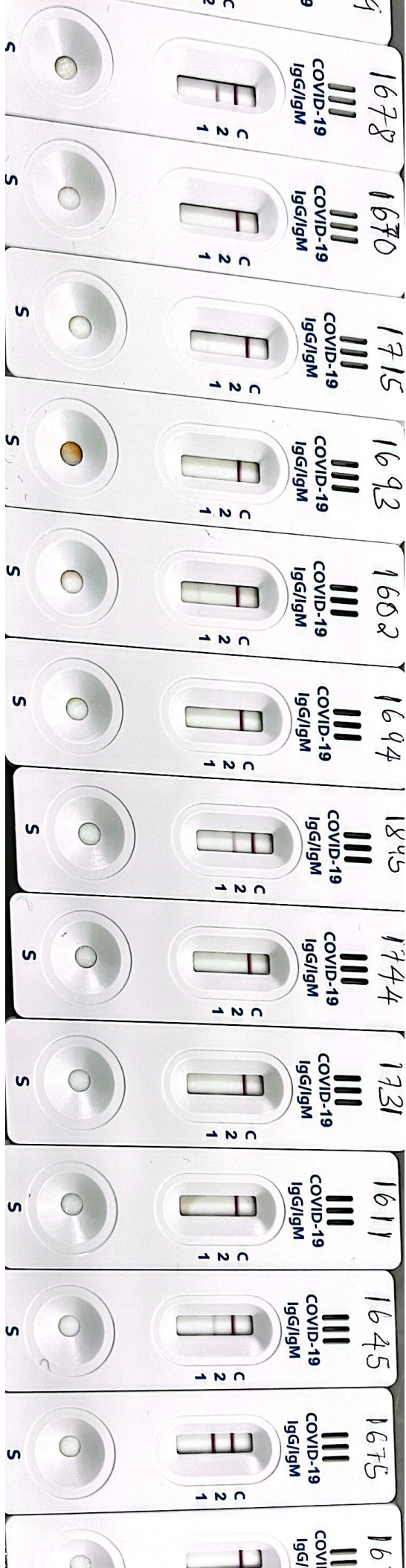

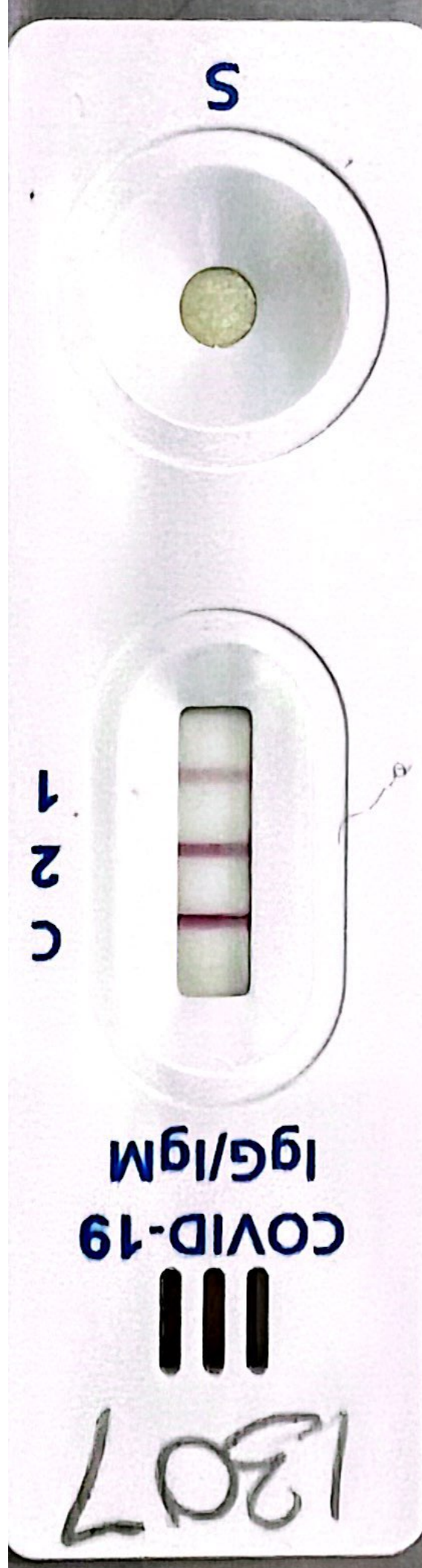

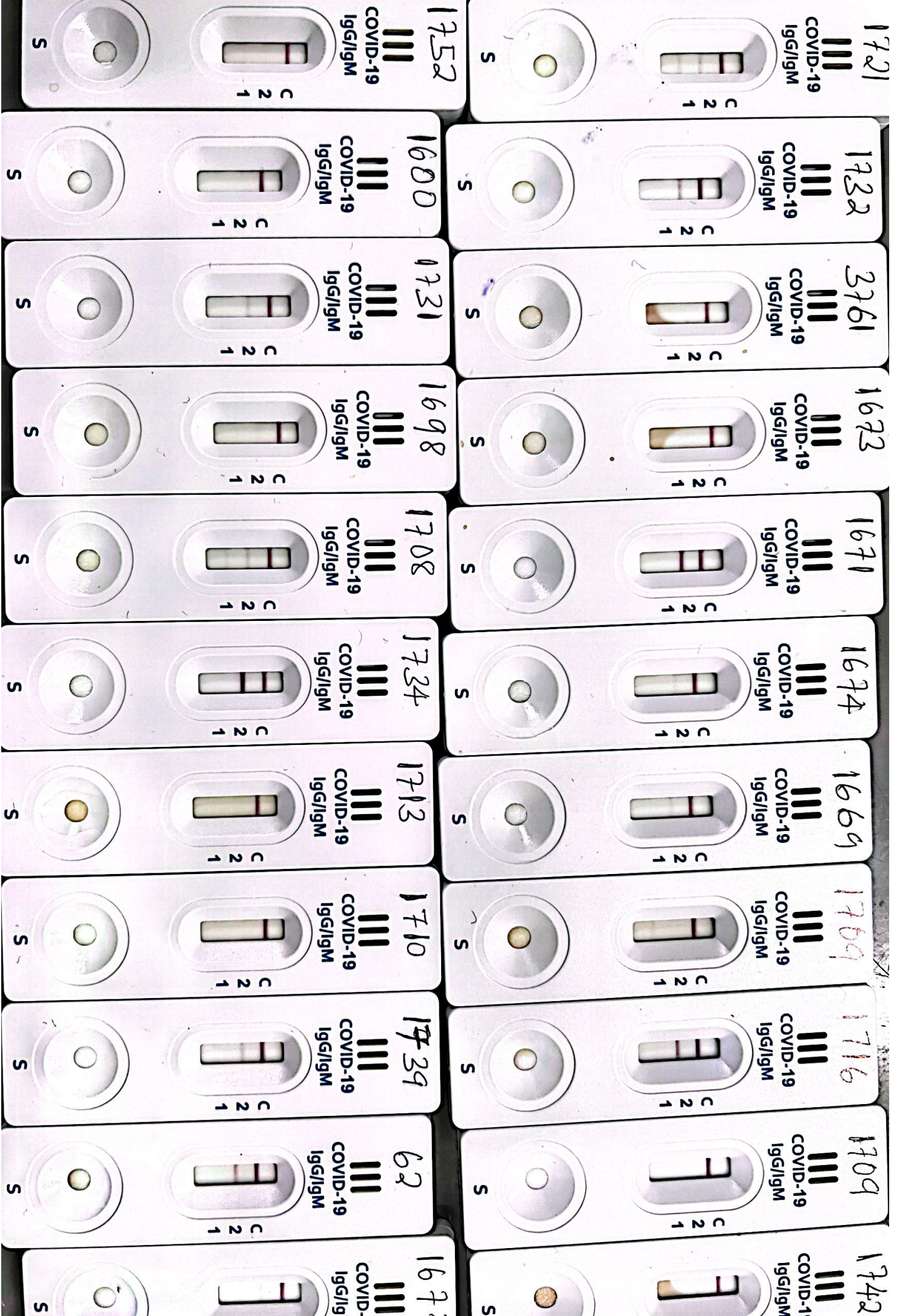

Supplement: Supplementary file 1 [file ijerph-19-11664-s001.zip › ijerph-1828929-supplementary.pdf]
